# Neuropilin-1 Modulates p53/Caspases Axis to Promote Endothelial Cell Survival

**DOI:** 10.1371/journal.pone.0001161

**Published:** 2007-11-14

**Authors:** Ling Wang, Shamit K. Dutta, Tatsuyoshi Kojima, Xiaolei Xu, Roya Khosravi-Far, Stephen C. Ekker, Debabrata Mukhopadhyay

**Affiliations:** 1 Department of Biochemistry and Molecular Biology, Mayo Clinic College of Medicine, Rochester, Minnesota, United States of America; 2 Department of Genetics, Cell Biology, and Development, University of Minnesota, Minneapolis, Minnesota, United States of America; 3 Department of Pathology, Harvard Medical School, Boston, Massachusetts, United States of America; University of Birmingham, United Kingdom

## Abstract

Vascular permeability factor/vascular endothelial growth factor (VPF/VEGF), one of the crucial pro-angiogenic factors, functions as a potent inhibitor of endothelial cell (EC) apoptosis. Previous progress has been made towards delineating the VPF/VEGF survival signaling downstream of the activation of VEGFR-2. Here, we seek to define the function of NRP-1 in VPF/VEGF-induced survival signaling in EC and to elucidate the concomitant molecular signaling events that are pivotal for our understanding of the signaling of VPF/VEGF. Utilizing two different *in vitro* cell culture systems and an *in vivo* zebrafish model, we demonstrate that NRP-1 mediates VPF/VEGF-induced EC survival independent of VEGFR-2. Furthermore, we show here a novel mechanism for NRP-1-specific control of the anti-apoptotic pathway in EC through involvement of the NRP-1-interacting protein (NIP/GIPC) in the activation of PI-3K/Akt and subsequent inactivation of p53 pathways and FoxOs, as well as activation of p21. This study, by elucidating the mechanisms that govern VPF/VEGF-induced EC survival signaling *via* NRP-1, contributes to a better understanding of molecular mechanisms of cardiovascular development and disease and widens the possibilities for better therapeutic targets.

## Introduction

Apoptosis of the endothelial cell (EC) has been suggested to play an important role in a number of common and life-threatening vascular diseases, such as atherosclerosis, hypertension, and restenosis [1]–[5]. EC apoptotic death-induced loss of EC number and EC dysfunction may constitute an initial causative step in, and have a critical role in, the progress of many vascular pathological situations by compromising vascular wall permeability to cytokines, growth factors, lipids and immune cells, increasing smooth muscle cell proliferation and enhancing blood coagulation [6]–[8]. However, the cascade of molecular events that precede these final outcomes is largely unknown. The intracellular signaling that regulates the onset and execution of apoptosis has only been partially elucidated [9], [10].

One possible reason for EC apoptosis may be the inappropriate function of growth factors and their receptors [11], which are critically involved in controlling cellular differentiation, growth, and function. One functionally relevant vascular growth factor is vascular permeability factor/vascular endothelial growth factor (VPF/VEGF) [12], [13] that has been cited as one of the most important pro-angiogenic factors. Neuropilin-1 (NRP-1) was recently found to be one of the VPF/VEGF receptors, which is expressed in EC and functions as an isoform-specific receptor for VPF/VEGF [14], [15].

Although the original evidence suggests that the function of NRP-1 in VPF/VEGF signaling in EC occurs *via* the formation of complexes involving VEGFR-2 and NRP-1 [14], [15], our findings and those of others have identified that NRP-1 regulates EC migration and adhesion to extracellular matrix proteins independently of VEGFR-2 [16], [17]. Moreover, recently published data by Pan *et al.* indicated that NRP-1 is crucial to modulating EC motility, thus demonstrating that it plays roles beyond acting as an enhancer of VEGFR2 signaling [18]. These observations suggest the possibility that NRP-1 may either interact with other signaling receptors or independently promote cell signaling. The latter is supported by our further studies in which we revealed that the C-terminal three amino acids of NRP-1 (SEA-COOH) can interact with neuropilin-1 interacting protein (NIP, also called RGS-GAIP-interacting protein <GIPC>), which mediates EC migration and angiogenesis [19]. Nevertheless, the molecular mechanisms of the downstream signaling of GIPC and its related function towards NRP-1 are not well understood.

Early in 1995, Alon *et al.* reported that VPF/VEGF could function as a potent inhibitor of apoptosis of ECs comprising newly formed retinal vessels in neonatal rats exposed to hypoxia [20]. Subsequently, Jain *et al.* reported VPF/VEGF could protect EC of newly formed immature tumor vessels [21]. Considerable progress has also been made towards delineating the VPF/VEGF survival signaling distal to the activation of VEGFR-2 [22]–[25]. However, a recent study found that the embryonic stem cell death pathway is overruled by a survival pathway during prolonged hypoxia (48 h) in a process involving HIF-1α-dependent up-regulation of VPF/VEGF which, in an autocrine action involving VEGFR-2 and NRP-1, protects against apoptosis [26]. Barr and co-workers [27] showed that NRP-1 plays an essential role in autocrine antiapoptotic signaling by VPF/VEGF in tumor cells and that an NRP-1-blockade with an anti-NRP-1 peptide corresponding to exon 7 [28] of VEGF165 induces tumor cell and EC apoptosis. However, a VEGFR-2-blockade only induces EC apoptosis [27], which suggests that both VEGFR-2 and NRP-1 play an important role in VPF/VEGF-mediated EC survival signaling. It also suggests that they may function together as a receptor complex. More interestingly, Bachelder's study found that NRP-1 supported VPF/VEGF autocrine function and further cell survival and chemotaxis in tumor cells lacking expression of VEGFR-1 and VEGFR-2 [29], [30]. To date, it is unknown whether NRP-1 can mediate VPF/VEGF-induced survival in EC independent of VEGFR-2 and the involved molecular signaling events.

By selectively activating NRP-1 in cell culture and by using a reverse genetic strategy in a zebrafish model, we explored the mechanisms that govern VPF/VEGF-induced EC survival signaling *via* NRP-1. We show here a novel mechanism for a VEGFR-2-independent, NRP-1-specific control of the anti-apoptotic pathway in EC which most likely involves GIPC and then is mediated by the activation, respectively, of PI3K/Akt, the p53/caspases and FoxOs axis, and p21.

### Results NRP-1 mediates VPF/VEGF-induced EC survival independent of VEGFR-2, and its C-terminal three amino acids are essential for this function

We first established a system to efficiently and reproducibly induce apoptosis in porcine aortic endothelial cell (PAEC) and human umbilical vein endothelial cell (HUVEC). Subconfluent cells were serum-starved (0.1%FBS) for 24 hours. Using TUNEL and Annexin V-FITC/PI apoptosis detection assays, we found that serum-starved cells showed a much higher prevalence of apoptosis and typical apoptotic nuclei (Figure S1, a, b) as compared to the cells grown in full medium.

**Figure 1:**
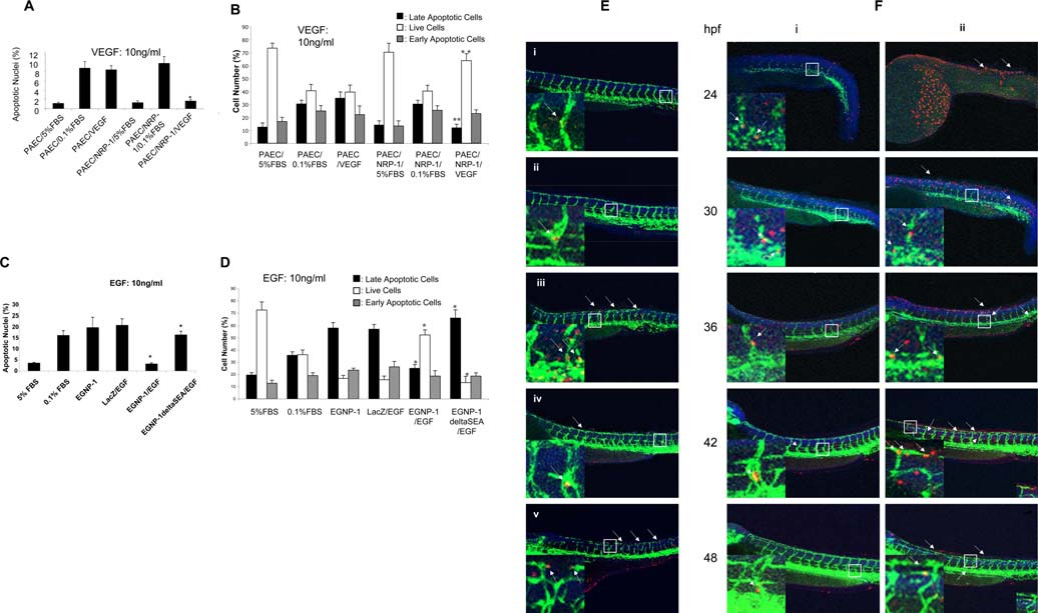
NRP-1 mediates VPF/VEGF-induced EC survival; the C-terminal three amino acids of NRP-1 are essential for this function. a. Quantitative determination of apoptotic nuclei in PAEC (n = 8). TUNEL assay was performed in apoptosis-induced PAEC and PAEC/NRP-1 with or with out 10ng/ml VPF/VEGF stimulation for 48 hrs to assess whether NRP-1 mediates PAEC survival. b. FACS apoptosis analysis to determine the percentage of apoptotic PAEC. c. Quantitative determination of apoptotic nuclei in HUVEC (n = 8. n: The number of counted field. All are same in this study). EGNP-1 mediates HUVEC survival; the C-terminal three amino acids of NRP-1 are essential for this function. TUNEL assay was performed in apoptosis-induced HUVEC transfected with EGNP-1 or EGNP-1ΔSEA and stimulated with or without 10ng/ml EGF. d. FACS apoptosis analysis to determine the percentage of apoptotic HUVEC. All the experiments at least were repeated three times and duplicated-wells were used for each experiment. *p<0.001, **p<0.005, ***p<0.01, ****p<0.05 in a Student's *t* test. e. NRP-1 mediates EC survival in zebrafish; the C-terminal three amino acids (SEA-COOH) are essential for the function. Fluorescent image of embryos injected with indicated morpholinos and mRNA, then subjected to the antibody stain with anti-GFP antibody and TUNEL assay to detect apoptosis. Insets show a magnification of apoptotic foci in the vessel. The arrows indicate apoptotic EC. i. Control; ii. Mismatch MO (4.5 ng); iii. zNRP-1a/1b MOs (4.5 ng+4.5 ng); iv. zNRP-1a/1b MOs (4.5 ng+4.5 ng)+hNRP-1 mRNA (0.3ng); v. zNRP-1a/1b MOs (4.5ng+4.5ng)+hNRP-1ΔSEA mRNA (0.3 ng). f. The dynamics of NRP-1-induced EC apoptosis in the zebrafish embryo. Fluorescent images of different stage embryos injected with indicated morpholinos and subjected to the antibody stain and TUNEL assay to detect apoptosis. Insets show a magnification of apoptotic foci in the vessel. The arrows indicate apoptotic EC. i. Control; ii. zNRP-1a/1b MOs (3.0 ng+3.0 ng).

As VEGFR-1, VEGFR-2, and NRP-1 are all expressed on EC, it is difficult to distinguish their biological functions and signaling pathways induced in EC by VPF/VEGF individually. Therefore, to elucidate the distinct roles of NRP-1, we first used PAEC that express none of the VPF/VEGF receptors. Wild-type PAEC and PAEC transfected with NRP-1 cDNA (PAEC/NRP-1) were analyzed for their survival when treated with VPF/VEGF. As shown in Supp. Fig. 1a, apoptotic cells of parental PAEC treated with VPF/VEGF were observed by TUNEL assay. However, VPF/VEGF induced a significant decrease in apoptosis in PAEC/NRP-1 relative to parental PAE cells, about five-and six-fold less than VPF/VEGF-stimulated parental PAEC or serum-starved PAEC/NRP-1 (Figure 1a). The percentage of apoptotic cells, which was determined using an Annexin V-FITC Apoptosis Detection Kit (Roche, Nutley, N.J.) with flow cytometry, demonstrated that the stimulation of PAEC/NRP-1 with VPF/VEGF significantly reduced apoptotic cell number, especially necrotic/late-apoptotic cells which were about three–fold lower than in VPF/VEGF-stimulated PAEC (Figure. 1b). This result is consistent with our findings using the TUNEL assay.

Furthermore, we engineered a chimeric receptor, EGNP-1, by replacing the extracellular domains of NRP-1 with the N-terminal domain of the epidermal growth factor receptor (EGFR), and another chimeric mutant receptor, EGNP-1?SEA (created by deleting the C-terminal three amino acids of NRP-1 (SEA-COOH)) [16].These receptors were used to further elucidate the role of NRP-1 in VPF/VEGF-induced EC survival and the importance of the C-terminal three amino acids of NRP-1 (SEA-COOH). The concomitant stimulation of serum-deprived EGNP-1-transduced HUVEC with epidermal growth factor (EGF, 10 ng/ml) led to a decrease in apoptosis more than five-fold compared to the non-stimulated control and the LacZ-transduced HUVEC. However, EGF stimulation in EGNP-1?SEA-transduced HUVEC did not yield a significant difference in apoptosis when compared to the control and to the LacZ-transduced HUVEC (Figure S1b, Figure 1c). Flow cytometry confirmed that EGF induced a significant decrease of apoptotic cells in EGNP-1-transduced HUVEC, but not in EGNP-1?SEA-transduced HUVEC, which, by contrast, showed more necrotic/late-apoptotic cells (Figure 1d). From these data, we concluded that NRP-1/EGNP-1 mediates ligand-induced EC survival independent of VEGFR-2, and that its C-terminal three amino acids (SEA-COOH) are essential for this function.

We employed a reverse genetic strategy to explore the function of NRP-1 in EC survival in a zebrafish model. Previously, we cloned zebrafish NRP-1 genes (zNRP-1a/1b) and analyzed their expression patterns by *in situ* hybridization [19]. In the current study, to examine functions of NRP–1 in zebrafish EC apoptosis, morpholinos are injected into a Fli-GFP transgenic fish line (TG(fli1:egfp)y1), where all of the ECs are labeled with GFP. Because of the transparency of zebrafish embryos, ECs can be observed in living embryos in fluorescent green. After that, the embryos were subjected to the TUNEL assay to detect apoptosis. The identity of the apoptotic cells is confirmed by using the red channel where EC should be labeled by Fli-GFP signal. The evaluation of apoptotic EC should consider the overlay of red signal and Fli-GFP green signal, and only red signal in EC location. As shown in Figure 1e and Figure S1, c, d, following knockdown of zebrafish NRP-1 by co-injecting NRP-1a/-1b splicing donor morpholinos (MO) into single-cell staged embryos, we found elevated levels of EC apoptosis compared with non MO injected or nucleotide exchange MO injection embryos. TUNEL-positive cells showed orange masses and were either rounded or shrunken (pycnotic). Furthermore, the specific EC apoptotic phenotype was suppressed by coinjection of human NRP-1 mRNA (Figure 1e, Figure S1, c, d). The ectopic expression of human NRP-1 in zebrafish efficiently blocked zebrafish NRP-1 knockdown-induced EC apoptosis, and the zebrafish showed normal vessels with established blood circulation (data not shown), and a normal EC phenotype (Figure 1e, Figure S1, c, d). In contrast, the human NRP–1 mRNA lacking its three C-terminal amino acids (SEA-COOH) failed to block zebrafish NRP-1 knockdown-induced vessel defects, nor did it abrogate EC apoptosis (Figure 1e, Figure S1, c, d). This suggests the functional importance of SEA-COOH in NRP-1-mediated EC survival.

We also investigated the dynamics of apoptosis in the zebrafish embryo. As shown in Figure 1f and Figure S1e, knockdown of NRP-1 revealed EC apoptosis as early as 24hpf embryos, which continued until 48 hpf. The early stage embryo had more apoptotic cells than the late stage embryo. Taken together, these results indicate that NRP-1 mediates EC apoptosis in zebrafish angiogenesis, and that its C-terminal three amino acids (SEA-COOH) are required for the function.

### GIPC conveys NRP-1-mediated EC survival signaling

**Figure 2:**
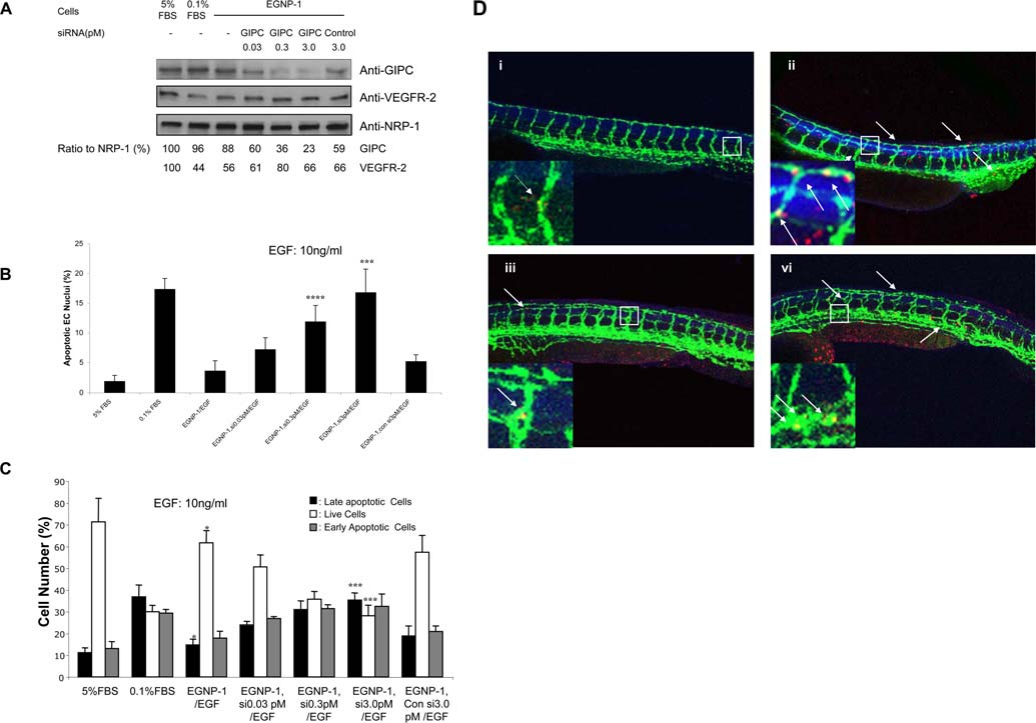
GIPC is involved in NRP-1-mediated EC survival. a. Knockdown of GIPC by siRNA in HUVEC. HUVEC were transfected with siRNA against GIPC in different concentrations. Immunoblotting was performed to quantify the expression levels of GIPC in these cells. GIPC and VEGFR-2 were quantified as a ratio to the amount of NRP-1. b. GIPC is involved in NRP-1-mediated HUVEC survival. Apoptosis assays were performed in apoptosis-induced HUVEC transfected with EGNP-1 and then transfected with siRNA, and followed by 10 ng/ml EGF stimulation for 48 hours. Quantitative determination of apoptotic nuclei (n = 8). Knock down of GIPC inhibits EGF-stimulated HUVEC apoptosis in a dosage-dependent manner. c. FACS apoptosis analysis to assess the percentage of apoptotic HUVEC. All the experiments at least were repeated three times and duplicated-wells were used for each experiment. *p<0.001, **p<0.005, ***p<0.01, ****p<0.05 in a Student's *t* test. d. GIPC is involved in NRP-1-mediated EC survival in zebrafish. Fluorescent image of embryos injected with indicated morpholinos and mRNA, then subjected to the antibody stain with anti-GFP antibody and TUNEL assay to detect apoptosis. Insets show a magnification of apoptotic foci in the vessel. The arrows indicate apoptotic EC. i. Control; ii. zGIPC MO (4.5 ng); iii. zGIPC (4.5 ng)+hGIPC mRNA (0.3 ng); iv. zGIPC MO (4.5 ng)+hGIPCΔPDZ mRNA (0.3 ng).

Based upon the above findings, we set out to further determine downstream pathways and the role of the involved key signaling molecules in NRP-1-mediated EC survival. Because our previous data showed that the C terminus of the RGS-GAIP (regulators of G-protein signaling-Gα interacting protein)-interacting protein, GIPC (also called NRP-1 interacting protein, NIP) can function as an adaptor protein to interact with the C-terminal three amino acids of NRP-1 (SEA-COOH) through its PDZ domain, and can then convey the NRP-1 signal to regulate EC migration and zebrafish angiogenesis [19], we first sought to address the role of GIPC in NRP-1-mediated EC survival. For this purpose, we knocked down GIPC in HUVEC using RNA interference-mediated silencing. After identifying that GIPC siRNA specifically and effectively silenced the expression of GIPC, but not that of NRP-1 or VEGFR-2 (Figure 2a), we then carried out apoptosis assays in HUVEC transduced with EGNP-1. We found that silencing of GIPC resulted in an increase in the percentage of apoptotic cells in HUVEC and also had marked morphological effects on the EC, including cytoplasmic shrinkage and cell retraction (Figure 2b, Figure S2a). To examine this result in more detail, we analyzed the percentage of apoptotic cells with flow cytometry. Indeed, we observed a notable difference in apoptosis between GIPC knock-down HUVECs and without GIPC knock-down HUVECs (Figure 2c). Necrotic/late-apoptotic and early apoptotic cells were significantly increased by increasing the amount of GIPC siRNA. These results were subsequently confirmed *in vivo* in our zebrafish model. Zebrafish GIPC morpholinos (1.5ng and 4.5ng) were injected into TG(fli1:egfp) embryos at the 1-4-cell stage. This not only disturbed the formation of the dorsal longitudinal anastomotic vessel (DLAV) and subintestinal vein (SIV) as described in our previous study [19], but also resulted in a significant increase in EC apoptosis, as revealed by TUNEL staining (Figure S2, b, c; Figure 2d). Additionally, circulation in the DLAV and SIV was diminished or blocked (data not shown). The body size was reduced as well (Figure 2d). These phenotypes resemble those from zNRP-1a/1b knockdown, which suggests that zGIPC is involved in the survival signaling pathway of NRP-1. Furthermore, we found that the GIPC-mediated EC apoptotic phenotype could be rescued with human GIPC mRNA (Figure S2, b, c; Figure 2d), whereas coinjection of hGIPCΔPDZ mRNA with the zGIPC morpholino did not rescue the zebrafish GIPC knockdown-induced EC apoptotic phenotype. In summary, these results suggest that GIPC is involved in NRP-1-mediated EC apoptosis, and that the PDZ domain of GIPC plays an important role in this function, probably by interacting with the C-terminal domain of NRP-1 (SEA-COOH).

### NRP-1-mediated EC survival requires PI3K/Akt activation

Since the survival signal mediated by various growth factors and cytokines may be dependent upon the phosphatidylinositol 3-kinase (PI3K)/Akt signaling pathway [31], [30], we investigated the possible regulatory role of PI3K/Akt in NRP-1-mediated survival.

**Figure 3:**
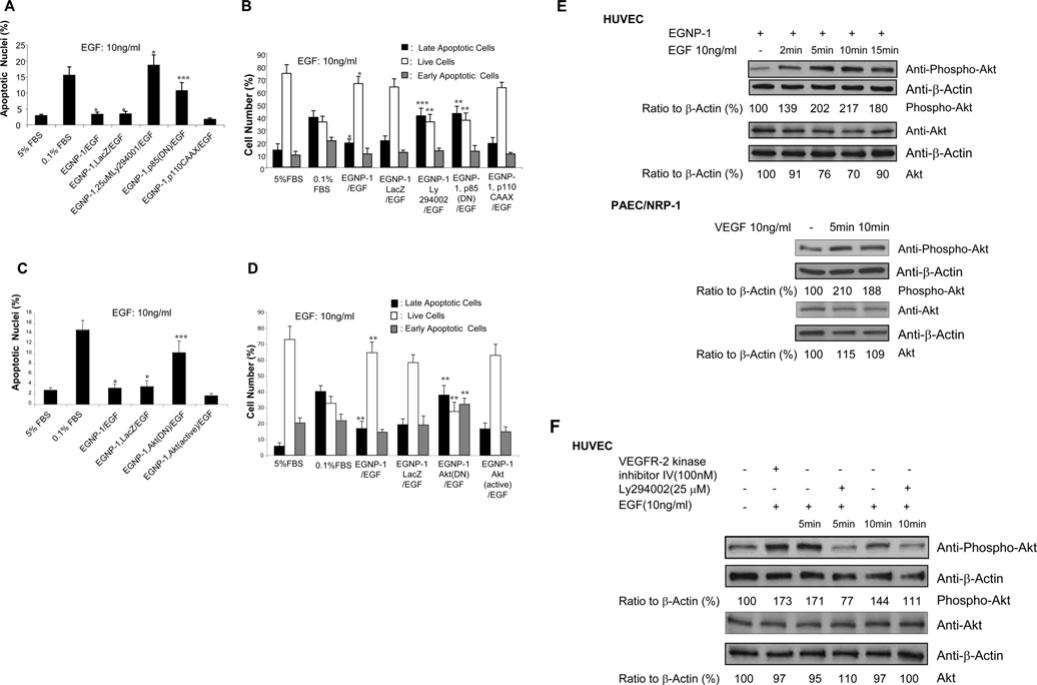
PI-3K/Akt is involved in NRP-1-mediated HUVEC survival. a. PI3K is involved in NRP-1-mediated EC survival signaling. Apoptosis assay was performed in apoptosis-induced HUVEC transfected with EGNP-1 or co-transfected with EGNP-1 and p85(DN) and stimulated with or without 10ng/ml EGF for 48hours, and then treated with LY294002 for 30 minutes, accordingly. Apoptotic nuclei were determined quantitatively (n = 8). b. FACS apoptosis analysis to quantify the percentage of apoptotic HUVEC. c. Akt is involved in PI3K-mediated NRP-1 survival signaling in EC. Apoptosis assays were performed in apoptosis-induced HUVEC transfected with EGNP-1 or co-transfected with EGNP-1 and Akt(DN) or Akt(Active) and stimulated with or without 10ng/ml EGF. Apoptotic nuclei were determined quantitatively (n = 8). d. FACS apoptosis analysis to determine the percentage of apoptotic HUVEC. All the experiments at least were repeated three times and duplicated-wells were used for each experiment. *p<0.001, **p<0.005, ***p<0.01 in a Student's *t* test. e. Akt phosphorylation by NRP-1/EGNP-1 activation. HUVEC were transfected with EGNP-1 and then stimulated with EGF. Immunoblotting with anti-phospho-Akt was carried out to quantify the expression levels of phospho-Akt in these cells. β-Actin was used as a loading control f. The influence of PI3K depletion and VEGFR-2 phosphorylation inhibition on EGNP-1-mediated Akt activation. Immunoblotting was performed in HUVEC transfected with EGNP-1 and then treated with Ly294002 or VEGFR-2 Kinase inhibitor IV. Phospho-Akt and Akt were quantified as a ratio to the amount of β-Actin

We first determined the role of PI3K in NRP-1-mediated EC survival signaling. As assessed by the number of apoptotic cells, after treatment of EGNP-1-transduced HUVEC with Ly294002 (a PI3K inhibitor, 25 µM) we observed a significant increase in HUVEC apoptosis (Figure 3a, Figure S3a), which suggests that blockage of PI3K almost completely impedes the survival effect of NRP-1. It is known that PI3K contains a kinase subunit (p110) and a regulatory subunit (p85) and functions in tyrosine kinase receptor signaling pathways [32]. The p85 adaptor subunit is known to form a constitutive heterodimer with the p110 catalytic subunit. Ligand-activated receptors interact with the p85 subunit, releasing the p110 subunit in an active form. In this study, we further used the dominant negative mutants of the p85 subunit (p85(DN)) and the constitutively activated mutant of PI3K (p110CAAX) to determine whether PI3K was required for EGNP-1-mediated EC survival. Our results showed that the co-transduced HUVEC expressed similar levels of EGNP-1 (data not shown), but the co-transduction of HUVEC with EGNP-1 and p85(DN) increased EC apoptosis compared to HUVEC co-transduced with EGNP-1 and LacZ (Figure 3a, Figure 3Sa). However, co-transfection of p110CAAX with EGNP-1 resulted in a slightly decreased level of EGF-induced, EGNP-1-mediated EC apoptosis. Flow cytometry also showed a similar result to that found with TUNEL: both Ly294002 and p85(DN) induced more EC apoptosis, especially late apoptosis (Figure 3b). These results indicate that the anti-apoptotic activity of NRP-1 on EC is mediated through PI3K.

It is known that the stimulation of growth factor receptor-induced PI3K activation mediates an increase in 3-poly-phosphoinositides, which in turn activates downstream effectors, such as the serine/threonine kinase Akt/protein kinase B (Akt/PKB) [32]–[34]. Therefore, we tested whether the PI3K effector protein Akt is involved in the survival response. We first co-transfected HUVEC with EGNP-1 and wild-type Akt or its two mutant forms, Akt(DN) (a dominant negative Akt mutant) and Akt(Active) (a constitutively activated Akt mutant), respectively. As revealed in Figure 3c and Figure 3Sb, Akt(Active) promoted EC survival to levels similar to those seen in EGNP-1 transduced HUVEC grown in the presence of EGF or 10% serum, whereas transfection of HUVEC with the dominant-negative Akt(DN) completely inhibited NRP-1-mediated EC survival activity. Flow cytometry confirmed the result showing significant high levels of late-and early-apoptotic cells (Figure 3d) in Akt(DN) co-infected HUVEC compared to Akt(Active) or LacZ co-infected HUVEC.

PI3k-dependent activation of Akt involves its phosphorylation at serine 473 [34]. To further verify that Akt is involved in NRP-1-mediated EC survival response, we analyzed whole-cell lysates of EGNP-1-transduced HUVEC treated with 10 ng/ml EGF for Akt activation, as indicated by phosphorylation at serine 473, using a phospho-specific antibody. In initial time course experiments, we found significant phospho-Akt upregulation as early as 2 minutes with maximal activation at 10–15 minutes after exposure of EGNP-1-transduced HUVEC to EGF (Figure 3e). The response gradually decreased after prolonged incubation (data not shown). As in HUVEC, the level of phospho-Akt was also upregulated in PAEC/NRP-1 stimulated by VPF/VEGF (Figure 3e).

Finally, we evaluated the effect of PI3K depletion on the activation of Akt. As shown by Western blot analysis, the PI3K inhibitor Ly294002 (25 µM) completely abolished Akt activation in response to EGF in EGNP-1-transduced HUVEC (Figure 3f), but the VEGFR-2 kinase inhibitor IV (100 nM), which can inhibit VEGFR-2 phosphorylation, did not influence EGNP-1-induced Akt phosphorylation (Figure 3f). These results suggest that Akt is a downstream effector in NRP-1-mediated EC survival signaling and exclude the possibility of involving VEGFR-2-mediated Akt activation in this study. In light of the above results, we conclude that PI3K/Akt is critically involved in NRP-1-mediated EC survival effects.

### p53 and the mitochondrial apoptotic pathway axis inactivation are involved in NRP-1-mediated EC survival

**Figure 4:**
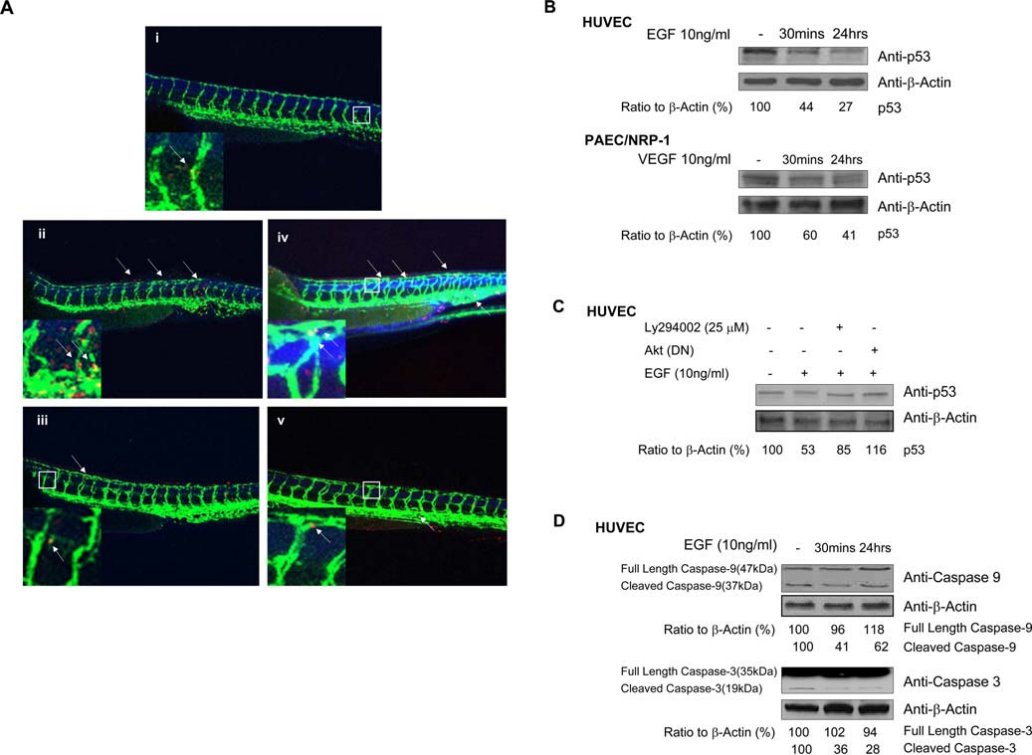
p53 inactivation in NRP-1-mediated EC survival. a. p53 is involved in NRP-1-mediated EC survival in zebrafish. Fluorescent image of embryos injected with indicated morpholinos and subjected to the antibody stain with anti-GFP antibody and TUNEL assay to detect apoptosis; Insets show a magnification of apoptotic foci in the vessel. The arrows indicate apoptotic EC. i. Control; ii. zNRP-1a/1b MOs (4.5 ng+4.5 ng); iii. zNRP-1a/1b MOs (4.5 ng+4.5 ng)+zp53 MO (9.0 ng); iv. zMdm2 MO (4.5ng); v. zMdm2 MO (4.5 ng)+zp53 MO (9.0 ng). b. NRP-1/EGNP-1-mediated p53 inactivation. EGNP-1 transduced HUVEC or PAEC/NRP-1 were subjected to EGF or VEGF for the indicated times and their lysates were immunoblotted for p53 with β-actin as a loading control. c. p53 is the downstream signaling molecule of PI3K/Akt in EGNP-1-mediated EC survival. HUVEC were transfected with EGNP-1 then treated with Ly294002 or co-transfected with EGNP-1 and Akt(DN). Immunoblotting was performed to determine p53 expression level. d. Inhibition of mitochondrial apoptotic pathway in EGNP-1-mediated EC survival. Immunoblotting was carried out in EGNP-1 transduced HUVEC stimulated with EGF at indicated times to assess the activation of caspase-9 and caspase-3. p53 and caspases were quantified as a ratio to the amount of β-Actin *p<0.001, **p<0.005, ***p<0.01, ****p<0.05 in a Student's *t* test.

About 13–18% of MOs used in zebrafish result in off-target effects [35], which are represented by a signature neural death peaking at the end of segmentation (1dpf, day post–fertilization). The embryos display small heads and eyes, somite and notochord defects and craniofacial defects. Collaborative information from Dr. Stephen C. Ekker (Department of Genetics, Cell Biology, and Development, University of Minnesota, Minneapolis) has shown that the off-target effects of MOs are mediated through p53-induced apoptosis. In this study, we found that knockdown of NRP-1a/-1b in zebrafish resulted in neural death phenotype and vessel defects, including EC apoptosis, suggesting that NRP-1 MOs may result in p53 activation. Alternatively, this may also suggest p53 is involved in NRP-1-mediated antiapoptotic effects. The latter seems more convincing in this study because we also observed that the morphant phenotypes from NRP-1 knockdown were rescued by human NRP-1 mRNA. It is quite likely that NRP-1 is specifically involved in cell death. For this purpose, Mdm2 MO and p53 MO co-injection embryos were used as controls. As we have shown previously, NRP-1 MOs induced vessel defects and EC apoptosis. In addition, we also observed tail and body axis, and neuron apoptosis (data not shown), that are very similar to the neural death phenotype induced by off-target effects of MOs. Interestingly, after co-injecting p53 MO with NRP-1a/-1b MOs, we found that knockdown of p53 mostly alleviated off-target neural death compared to the positive control, while the vessel defects by knockdown NRP-1 were also mostly rescued (Figure 4a, Figure S4, a, b). These results suggest that p53 is involved in NRP-1-mediated EC survival.

This finding was further confirmed by investigating the effect of NRP-1 activation on p53 expression. By stimulating EGNP-1-transduced HUVEC with 10 ng/ml EGF, p53 protein expression levels decreased significantly at 30 minutes to 24 hours when compared to the control group without EGF stimulation (Figure 4b). This indicates that the EGNP-1-mediated EC antiapoptotic effect is induced by inhibition of p53 activation. Similar results for p53 expression level were also observed in PAEC/NRP-1 (Figure 4b). These results support our *in vivo* findings.

A previous study reported that effective recruitment of Akt by appropriate survival signals may lead to activation of Mdm2, inactivation of p53, and eventually inhibition of p53-dependent apoptosis [36]. We, therefore, further investigated whether p53 is downstream of PI3K/Akt in NRP-1 signal. As shown in Figure 4c, the inhibition of PI3K with Ly294002 or blockage of Akt with the dominant negative mutant of Akt (Akt(DN)) reversed the expression of p53 to a higher level in EGNP-1-transduced HUVEC stimulated with EGF, which suggests that p53 is, in fact, downstream of PI3K/Akt in NRP-1-mediated EC survival.

Caspases are involved in both the mitochondria-independent (extrinsic) and the mitochondria-dependent (intrinsic) pathways [37], [38], the two main pathways of cell death signaling in biological systems. p53 can regulate the secretion of cytochrome c to initiate the mitochondria-dependent pathway, leading to the activation of caspase-9 then caspase-3 followed by apoptosis [39]. p53 also regulates a series of genes that initiate the mitochondria-independent pathway, resulting in caspase-8 and -3 activities and apoptosis [40]. For these reasons, we sought to determine whether NRP-1's anti-apoptosis is associated with inactivation of caspases. We examined the cleavage of the key apical caspases in the extrinsic pathway (caspase-8) and in the intrinsic pathway (caspase-9) as well as the cleavage and activity of caspase-3, which is the major executioner protease for both pathways. Cleavage of caspase zymogens is instrumental to the activation of caspase-3 and directly follows proximity-induced activation of apical caspases, thus contributing to their sustained activity [37], [41]. Pro-caspase-9 is the apical caspase of the intrinsic pathway and is efficiently processed within the apoptosome [37]. In this study, cleavage of pro-caspase-9 was induced by serum starvation for 24 hours in EGNP-1-transduced HUVEC. Western blot analysis showed the full length caspase-9 (47 kDa) and a cleaved fragment (37 kDa) (Figure 4d). Following 30 minutes to 24 hours of stimulation with EGF in these cells, we found that the levels of cleaved caspase-9 decreased, as indicated by the concomitant increase of the pro-caspase band and the decreased formation of the p35 fragment (Figure 4d). Activated or cleaved caspase-3 (17 and 19 kDa) is crucial for the induction of apoptosis. After 24 hours serum starvation, pro-caspase-3 is proteolytically cleaved to generate a p19 signature fragment in EGNP-1-transduced HUVEC, but the cleavage level decreased following EGF stimulation (as shown in Figure 4d). In this study, cleavage of caspase-8 was not detected (data not shown). These results indicate that sustained activation of NRP-1 inhibits caspase-9, and consequently directly suppresses the cleavage of its preferred substrate, caspase-3, therefore may reduced EC apoptosis. Taken together, the above results confirm that the NRP-1-mediated EC anti-apoptotic affects occur through the axis inactivation of p53 and mitochondrial apoptotic pathway.

### Induction of p21 is involved in NRP-1-mediated EC survival

**Figure 5:**
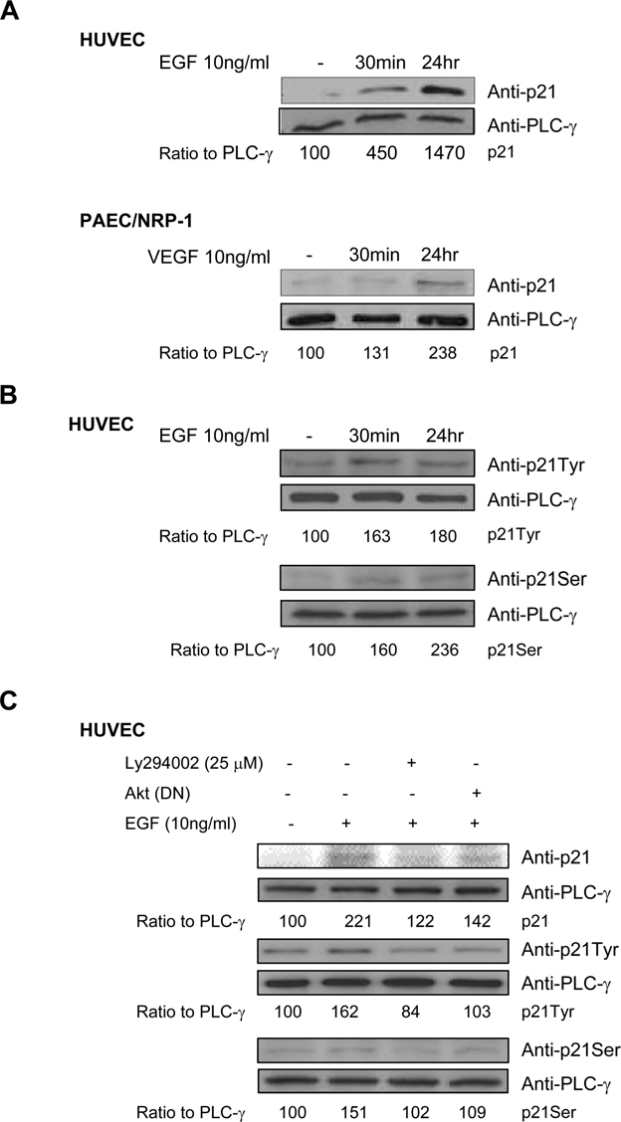
Induction of p21 through PI3K/Akt is involved in the survival signaling of NRP-1. a. The activation of NRP-1/EGNP-1 increased p21 expression. EGNP-1 transduced HUVEC or PAEC/NRP-1 were subjected to EGF or VEGF for the indicated times and their lysates were immunoblotted for p21 with PLC-γ as a loading control. b. The activation of EGNP-1 induced p21 phosphorylation at its Tyr^145^ and Ser^146^ sites. Immunoblotting was performed with anti-p21Tyr and anti-p21Ser in EGNP-1 transduced HUVEC stimulated by EGF for the indicated times. c. Akt mediates the phosphorylation of p21 in EGNP-1-mediated signaling. HUVEC were transfected with EGNP-1 then treated with Ly294002 or co-transfected with EGNP-1 and Akt(DN). Immunoblotting was performed to quantify the expression and activation of p21. p21 and phospho-p21 were quantified as a ratio to the amount of PLC-γ. *p<0.001, **p<0.005, ***p<0.01, ****p<0.05 in a Student's *t* test.

p53 is a transcription factor, and its loss results in changes in the expression of downstream target genes. One such target gene is p21^Cip1/WAF1^(p21), which we examined in this study. As shown in Figure 5a, we observed a significant increase in p21 protein levels from 30 minutes to 24 hours after EGF stimulation in EGNP-1-transduced HUVEC or VPF/VEGF stimulation in PAEC/NRP-1. Obviously, this increased p21 expression level is independent of p53 inhibition. The cyclin kinase inhibitor p21 was initially believed to be an inhibitor of cell cycle progression, but has since been shown to have additional functions other than CDK inhibition. A recent study reported that AKT/PKB phosphorylation of p21 enhances the protein stability of p21 and promotes cell survival [42]. Based on this evidence, we first examined whether NRP-1 activation can induce p21 phosphorylation. We used two antibodies against the potential AKT/PKB phosphorylation sites of p21, Thr^145^ and Ser^146^. Figure 5b demonstrates that p21 is phosphorylated by EGNP-1 in the two positions. Furthermore, we tested whether the increased expression level of p21 results from NRP-1-mediated PI3K/AKT activation. Indeed, our results showed that there was a reduced expression level of p21 in LY294002 treated or Akt(DN) co-infected HUVEC, compared to the control HUVEC infected only with EGNP-1. Meanwhile, we found that the phosphorylation levels of p21 at sites Thr^145^ and Ser^146^ were also reduced (Figure 5c). Together, these data suggest that NRP-1-mediated EC survival occurs through Akt/PKB activation-induced phosphorylation and up-regulation of p21.

### Modulation of FoxO transcription factor activation in NRP-1-mediated EC survival signaling

**Figure 6:**
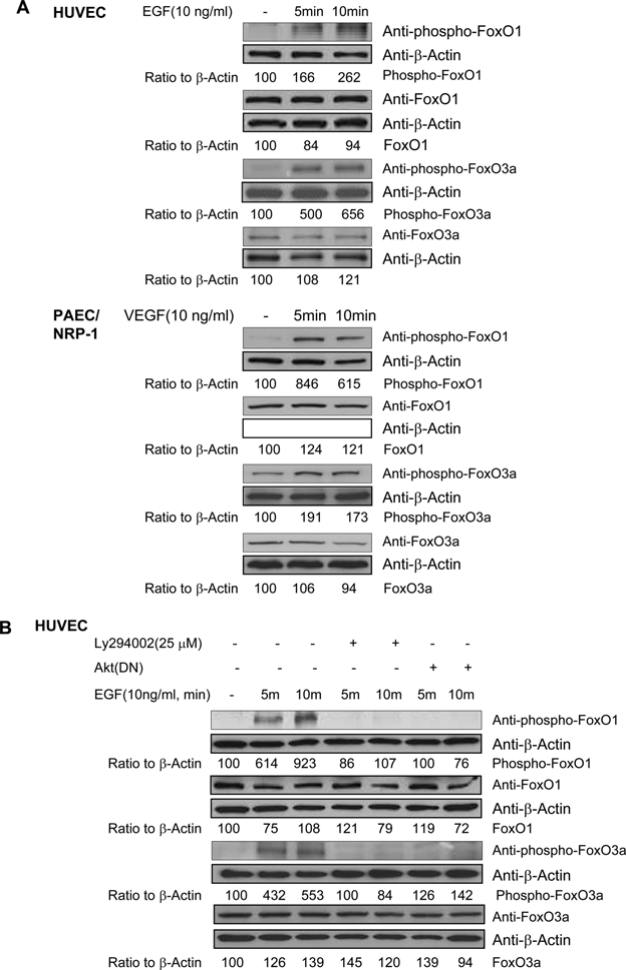
Involvement of transcription factors FoxO1 and FoxO3a in NRP-1-mediated EC survival signaling. a. Phosphorylation of FoxO1 and FoxO3a in NRP-1 survival signaling. After EGF stimulation of EGNP-1-transduced HUVEC or VEGF stimulation of PAEC/NRP-1 for the indicated times, the protein levels in cell extracts of FoxO1 and FoxO3a were assessed by immunoblotting. b. PI3K/Akt depletion resulted in reduction of FoxO1 and FoxO3a phosphorylation. HUVEC were transfected with EGNP-1, then treated with Ly294002 or co-transfected with EGNP-1 and Akt(DN). Immunoblotting was performed to determine the phosphorylation level of FoxO1 and FoxO3a with β-Actin as a loading control. phospho-FoxOs and FoxOs were quantified as a ratio to the amount of β-Actin. *p<0.001, **p<0.005, ***p<0.01, ****p<0.05 in a Student's *t* test.

Studies have suggested that PI3K-mediated Akt activation in mammals negatively regulates the Forkhead box class O (FoxO) subfamily of Forkhead transcription factors [43], [44]. Akt mediates phosphorylation of FoxO proteins [44], [45], which prevents their nuclear translocation, thereby inhibiting their transcriptional competence. Studies in mammalian cells have shown that the overproduction of FoxO1 (FKHR), FoxO3a (FKHRL1), and FoxO4 (AFX) induces either cell cycle arrest or apoptosis [44], [46]. Based upon these findings, we decided to address whether PI3K/Akt-mediated NRP-1 survival signaling occurs through inhibition of FoxO transcription factors. HUVEC are known to express FoxO1 and FoxO3a, whereas FoxO4 could not be detected [47]. We therefore focused on these two FoxO transcription factors. As we expected, Western blot analysis showed that 10ng/ml EGF or VPF/VEGF stimulation resulted in a significant increase in the phosphorylated forms of FoxO1a and FoxO3a in the EGNP-1-transduced HUVEC or PAEC/NRP-1 (Figure 6a). Furthermore, depletion of PI3K or blockage of Akt resulted in a significant decrease in the phosphorylated forms of FoxO1 and FoxO3a proteins in EGNP-1-transduced HUVEC (Figure 6b). Our results suggest that the survival signaling of NRP-1 in EC is mediated by PI3K/Akt through the inhibition of FoxO1 and FoxO3a.

## Discussion

In this study, we show a novel function for NRP-1, which can mediate VPF/VEGF-induced EC survival independent of VEGFR-2. We used PAEC transfected with NRP-1 cDNA to examine the specific function of NRP-1. PAEC/NRP-1 was a generous gift of by Dr. Shay Soker (Molecular and Cell Biology, Wake Forest Institute for Regenerative medicine). Previously, Soker *et al.* established stable cell lines synthesizing neuropilin-1 (PAEC/NRP-1) and characterized VEGF165 binding to NRP-1 in the cell [48]. PAEC were chosen for this study due to their lack of expression of any VPF/VEGF receptors [49]. To explore the possibility that NRP-1 might transduce VPF/VEGF signaling alone, NRP-1 must be selectively activated, since VPF/VEGF receptors VEGFR-1 and VEGFR-2 are also expressed in EC. To do so, we used the chimeric receptor EGNP-1 by replacing the extracellular domains of NRP-1 with the N-terminal domain of EGFR. At ≤80% confluence, early passage HUVEC doesn't express EGFR, and therefore, the fusion receptor can be used to distinguish the signaling pathway that is mediated by NRP-1 [16]. This might seem to be scant evidence because NRP-1 might form a complex with VEGFR-2 through its intracellular domain. However, previously using EGNP-1, together with co-transducing HUVEC with EGDR mutants or blocking VEGFR-2 with an anti-VEGFR-2 antibody, we demonstrated that NRP-1 mediates HUVEC migration independent of VEGFR-2 [16]. This suggests that NRP-1 can indeed function independently of VEGFR-2 in VPF/VEGF signaling and supports our findings in this study. The current understanding of NRP-1 function is primarily based upon studies of its extracellular domain. However, the chimeric receptor EGNP-1, though it lacks the extracellular domain of NRP-1, has been shown to be involved in EC survival. This hints there may be a distinguishing pathway whereby NRP-1 mediates EC survival through its intracellular domain and is independent of VEGFR-2 signaling. Indeed, our published data have shown that the C-terminal three amino acids of NRP-1 (SEA-COOH) can interact with GIPC and are involved with NRP-1-mediated EC migration [19], whereas we failed to identify VEGFR-2 in the complex of NRP-1 and GIPC (data not shown). These facts, together with the observation in the present study that the C-terminal three amino acids and GIPC are essential for NRP-1-mediated EC survival, support the existence of an independent signaling pathway for NRP-1-mediated VPF/VEGF function. Overall, these results indicate that NRP-1 can mediate VPF/VEGF-dependent EC survival as an independent receptor.

Our previous study demonstrated that GIPC functions as a key adaptor protein that is involved in NRP-1-mediated EC migration and angiogenesis [19]. Similarly, we found that knockdown of GIPC in this study suppresses NRP-1-mediated anti-apoptotic effects in HUVEC and increases EC apoptosis in the zebrafish model, suggesting that GIPC addresses the same signaling pathway as NRP-1 in NRP-1-mediated EC survival. Consequently, how GIPC conveys NRP-1 survival signaling in EC is obviously an important issue that should be targeted.

In this study, we found that the PI3K inhibitors Ly294002 and p85(DN), a dominant negative mutant of the p85 subunit, reduced levels of NRP-1/EGNP-1-dependent survival. These observations indicate that PI3K is involved in NRP-1-mediated EC survival signaling, which is consistent with the findings by Bachelder *et al.* that NRP-1 supported VPF/VEGF autocrine function and promoted breast carcinoma cell survival by stimulating the PI3K pathway [29]. Activation of PI3K phosphatidylinositol-3, 4, 5-triphosphate positively regulates Akt by binding to the pleckstrin homology domain of Akt [50]. In our experiments we found that Akt phosphorylation levels increased after EGF stimulation of EGNP-1-transduced HUVEC, and that such activation could be inhibited by the PI3K-specific inhibitor Ly294002 but not influenced by inhibiting VEGFR-2 phosphorylation. This confirms that NRP-1 survival signaling induces PI3K-dependent Akt phosphorylation. The ability of activated Akt to promote survival has been found in fibroblasts [51] and heochromocytoma cells [52] and neuronal cells [32]. In accordance with these findings, we found that the kinase-inactive Akt mutant (Akt(DN)), that has been suggested to act in a dominant-negative manner [53], blocks NRP-1-mediated survival activity on EC. This finding constitutes the further demonstration of a role played by the PI3-kinase/Akt pathway in mediating a NRP-1 survival activity. Despite the additional information provided by the above results, how PI3K is activated in NRP-1 survival signaling still remains unknown. p85, the regulatory subunit of PI3K, contains two SH2 domains and a SH3 domain. The SH2 domain of p85 is responsible for interaction with phosphorylated tyrosine in the context of motif YXXM [54], [55]. However, unlike VEGFR-2, the intracellular domain of NRP-1 lacks the YXXM motif. Since GIPC is a central adapter protein for NRP-1 signaling, it is tempting to speculate that GIPC may function as an adaptor molecule, mediating the activation of p85 by NRP-1, which is currently under investigation.

Given that a transformed approach might alter signaling pathways (Dr. Stephen C. Ekker, Department of Genetics, Cell Biology, and Development, University of Minnesota, Minneapolis), especially those that regulate cell survival, the involvement of p53 in regulating the apoptotic pathway suppressed by survival signals from NRP-1 was addressed more comprehensively by both *in vivo* and *in vitro* studies in this work. In the zebrafish model, EC apoptosis and vessel defects resulting from the loss of NRP-1 are recovered when p53 expression is downregulated. This suggests that p53 acts upon downstream components of a NRP-1 activation-induced survival cascade. The p53 pathway is composed of hundreds of genes and their products that respond to a wide variety of stress signals, including apoptosis and cell cycle arrest. The former involves caspase cascade while the latter is mediated by p21. Decreased levels of p53 might suppress caspase cleavage and therefore downregulate apoptosis. Indeed, our *in vitro* data found that activating NRP-1 decreases the expression of p53, caspase-9 and caspase-3, which confirms our *in vivo* observations. Our findings that NRP-1-induced cell survival more directly affects caspase-9, then caspase-3, but not caspase-8 cleavage, also supports the concept that phospholipases may be primarily involved in the intrinsic (mitochondria-dependent) arm of the bifurcate pathways of apoptosis mediated by death receptors. In addition, the data shown here that p53 is the downstream signaling molecule of PI3K/Akt hints that Mdm2 may be another considerable signaling component in NRP-1 survival signaling in EC, thereby connecting connects PI3K to p53.

p21 was originally isolated as a p53-inducible protein and identified as an inhibitor of cyclin-dependent kinases (CDKs), and thus thought to function as a critical regulator of the cell cycle [56]–[58]. However, a number of previous studies suggested that p21 levels were alternatively regulated by p53-independent mechanisms. Serum and other growth factors might be involved with the up-regulation of p21 levels in various cell types [59], [60]. The activator of the PI3K pathway, EGF, increased p21 levels by increasing the half-lives of both transcript as well as protein [61]. Additionally, the PI3K pathway was involved with the modulation of p21 levels [62], [63]. Inhibitors of the PI3K pathway reduced the level of p21 in both cases. Moreover, recent investigations have clearly demonstrated that p21 also plays a role in allowing cell cycle transit as well as preventing apoptosis. Many late stage, rapidly proliferating tumors show a profound elevation in the levels of p21, which is counterintuitive in light of the proposed inhibition of cell cycle progression mediated by p21 [64]-[66]. The pro-survival activity of p21 in cells treated with chemotherapeutic agents such as taxol also argues for a role for this protein in the protection of tumors *in vivo* [67], [68]. The similar pro-survival effect is also observed in terminally differentiated cells such as myoblasts [69], hematopoietic stem cells [70], and macrophages [71]. An exciting finding shown by Li *et al.*, indicated that Akt phosphorylation of p21 at sites ^145^ and Ser^146^ resulted in increased p21 stability as well as enhanced glioblastoma cell survival [42]. Notably, the results from our current study are consistent with these observations, confirming a crucial role for p21 as an anti-apoptotic protein, and also providing evidence that p21 levels can be regulated directly by PI3K/Akt. In addition, the elevated levels of p21 may also partly result from activated PI3K/Akt-induced caspase-3 inactivation, which reduces caspase-3-mediated p21 cleavage. This is consistent with a previous study that caspase-3-mediated cleavage of p21 during the apoptotic process sends growth-arrested cells to apoptosis[72]. While this novel role of p21 is evolving, a recent study revealed that p21 blocks irradiation-induced apoptosis downstream of mitochondria by inhibition of cyclin-dependent kinase-mediated caspase-9 activation [73]. Since the mechanisms by which p21 interferes with the death machinery are still largely unknown, however, this remains our current focus.

FoxO transcription factors are key regulators of cell fate. The mammalian FoxO family of transcription factors FoxO1, FoxO3a, and FoXO4 are major substrates of the protein kinases PKB (Akt). Activation of the PI3K pathway blocks the function of all 3 FoxO members by Akt-dependent phosphorylation of three conserved residues, which relay PI3K signals to target genes [74], [75]. A recent study has shown that FoxO1 and FoxO3a are the predominant FoxO isoforms expressed in mature EC [47]. In this study, we found that NRP-1 induced the rapid phosphorylation of FoxO1 and FoxO3a and was mediated by activated PI3K/Akt. These data suggest a requirement for FoxO1 and FoxO3a inactivation during NRP-1-induced EC survival and are consistent with previous studies into the roles of FoxO1 and FoxO3 in the induction of cell apoptosis [44], [76]. The mechanism whereby FOXO proteins promote apoptosis in different cell types appears surprisingly diverse and includes induction of death-receptor ligands (*e.g.* FasL) and proapoptotic factors (*e.g.* Bim) as well as repression of cytoplasmic (*e.g.* c-FLIP) and mitochondrial antiapoptotic proteins (*e.g.* Bcl-2 and Bcl-xL) [77]–[80]. How FoxO1 and FoxO3a function in PI3k/Akt-dependent NRP-1 survival signaling in EC has not been fully defined in this study and remains to be elucidated.

**Figure 7:**
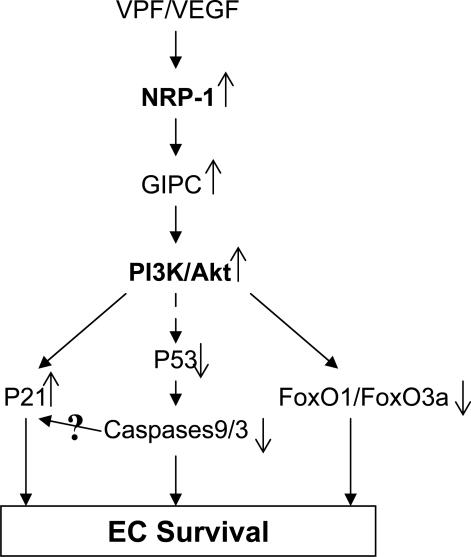
The working model of NRP-1-mediated EC survival. NRP-1 mediates VPF/VEGF-induced EC survival effects in EC by connecting NRP-1 and GIPC to activation of PI3k/Akt and subsequent inactivation of p53 pathways and FoxOs, as well as activation of p21.

In summary, after finding that NRP-1 can mediate VPF/VEGF-induced EC survival independent of VEGFR-2, our results also highlight, for the first time, the distinct signaling pathways of NRP-1 in VPF/VEGF-induced survival effects in EC by connecting NRP-1 and GIPC to activation of PI3k/Akt and subsequent inactivation of p53 pathways and FoxOs, as well as activation of p21. Based upon these findings, we proposed the working model in detailed in Figure 7.

What is emerging from this study, also in the light of reports from our previous studies and others, is that NRP-1 can function alone to mediate VPF/VEGF signaling. The consistency of our results supports the two current hypotheses for NRP-1 function in VPF/VEGF signaling in EC: either that NRP-1 is involved in VPF/VEGF-induced EC function by acting as a VEGFR-2 co-receptor, or alternatively, as a functional receptor alone. Not only this, recognizing the mechanistic aspects of coreceptor-based or single receptor–based signaling ligand guidance cues may have implications for other ligand guidance and complicated receptor-mediated events which form the basis of key biological functions. In addition, the multiple pathways involved in NRP-1-mediated VEGF survival signaling indicate a degree of complexity that is paralleled by the emerging complexity of interaction between VEGF ligands and their receptors, and the downstream signaling pathways they mediate.

To the best of our knowledge, both the VEGFR-2 signal alone, as well as NRP-1 acting in concert with VEGFR-2, are involved in VPF/VEGF-induced EC survival *in vivo*. As a consequence, NRP-1 signaling alone only partly determines EC fate. This implies that an understanding of the role of NRP-1 only provides part of the picture of VPF/VEGF survival signaling. However, given our increasing understanding of NRP-1, we can generate a more complete scenario of how the signaling molecule determines cell behavior.

## Materials and Methods

### Materials

Human EGF, human VEGF, primary HUVEC, EGM-MV bullet kit and EC basic medium (EBM) were purchased from CLonza (San Diego, CA). F12 Medium was purchased from GIBCO (Carlsbad, CA). *Optimem medium and lipofectamine 2000 were purchased from Invitrogen (Carlsbad, CA).* GIPC siRNA was purchased from Santa Cruz Biotechnology, Inc. (Santa Cruz, CA). The following antibodies were used: Goat polyclonal antibody against the NRP-1-C-terminal domain, anti-GIPC, anti-p53 and anti-p21 were purchased from Santa Cruz Biotechnology, Inc. (Santa Cruz, CA); anti-Akt1/PKB, anti-phospho-Akt1/PKB(Ser473), anti-FoxO3a (FKHRL1), and anti-phospho-FoxO3a (FKHRL1, Ser253), were purchased from Upstate Biotechnology (Waltham, MA); anti-FoxO1a (FKHR), anti-phospho-FoxO1a (FKHR, Ser256), anti-cleaved caspase-3, anti-caspase-8, and anti-caspase-9 were purchased from Cell Signaling Technology (Beverly, MA); and anti-ß-actin was purchased from Sigma Chemical Company (St. Louis, MO). Anti-GFP antibody was purchased from Invitrogen. The quantification of protein was performed with a digital analysis of protein bands (TIFF images) using UN-SCAN-IT software (Silk Scientific, Orem, UT). VEGFR-2 Kinase Inhibitor IV was purchased from Calbiochem (San Diego, CA).

### Cell culture and treatment

Parental PAEC and PAEC expressing NRP-1 (PAEC/NRP-1) [were kindly provided by Dr. Shay Soker (Molecular and Cell Biology, Wake Forest Institute for Regenerative medicine) and were grown in F12 medium containing 10% FCS and GPS[48]. HUVEC (CLonza, San Diego, CA) were cultured as described previously. HUVEC were grown on 30 µg/ml vitrogen-coated dishes using the EGM-MV Bullet Kit (5% fetal bovine serum [FBS] in EBM with 12 µg/ml bovine brain extract, 1 µg/ml hydrocortisone, and 1 µg/ml GA-1000).

HUVEC (passage 3 or 4) that were ∼80% confluent were used for most experiments. Apoptosis was induced by deprivation using EBM supplemented with 0.1% BSA for 24 h. After that, cells were exposed to 10ng/ml VPF/VEGF or EGF in the same medium for 48 hours and then used for apoptosis testing.

### Zebrafish lines and maintenance

Zebrafish were maintained and bred as described previously[81] (All experiments were performed using the TG(fli1:egfp)y1 l transgenic line that has been described previously[82] (Embryos were staged according to Kimmel *et al* [83].

### TUNEL assay

Detection and quantification of EC apoptosis was performed using an In Situ Cell Death Detection Kit, TMR red Roche, Nutley, NJ). The assay was performed following the manufacturer's protocol. Samples were dechorionated and fixed in 4% paraformaldehyde for 1 hour at room temperature. Then they were washed with PBS twice and permeabilized with 0.1% sodium citrate, 0.1% TritonX for 2 minutes on ice. After washing twice in PBS, samples were incubated with the reaction mixture containing the terminal deoxynucleotidyl transferase and TMR labeled nucleotides for 1 hour in the dark at 37°C. The reaction was stopped by washing with PBS three times. Terminal deoxynucleotidyl transferase catalyzed the incorporation of labeled nucleotides to 3-OH DNA and ends in a template-independent reaction. The fluorescent signal was visualized and imaged using a Zeiss Axioplan 2 microscope coupled to an Apotome, using AxioVision 4.2 software (Zeiss). Z-stacks were superimposed using Extended Focus feature of the software.

### Flow cytometry analysis

Cells (5×10^4^) were seeded on the slide cover in six-well culture plates and treated for 48 h with VPF/VEGF (10 ng/ml) in medium containing 0.1% FBS. Cells were washed with PBS and apoptosis was assessed using the Annexin V-FITC/PI apoptosis detection kit (BioVision, Inc., Mountain View, CA) according to the manufacturer's instructions. The respective proportions of apoptotic cells and necrotic/late-apoptotic cells were measured on 20,000 cells with a FACS Calibur analysis system (Becton Dickinson, CA, USA).

### RNA interference

HUVECs at 70% confluency will be transfected in optimem medium with the siRNA duplexes using lipofectamine 2000 (Invitrogen). After 4 hours the transfection medium will be removed, the cells will be washed twice with PBS and then maintained in complete HUVEC medium for 48 hours before performing experiments with VPF/VEGF stimulation as above.

### Morpholino gene knock-down in zebrafish

Morpholinos were designed targeting splice donors of the specific genes to efficiently block pre-mRNA splicing. Zebrafish NRP-1a/NRP-1b MOs, p53 MO, and MDm2 MO were designed as described. We purchased Morpholino antisense oligonucleotides from Gene Tools, Inc, (Philomath, OR). The sequences are available upon request. The injection solution was prepared as described. Morpholino solutions with 3.0 and 6.0 ng MOs were injected into zebrafish embryos at the 1-4 cell stage, respectively. Embryos were examined for EC apoptosis and vascular defects at different time points. Quantitative RT-PCR (Ambion, Austin, TX) was used to monitor the splicing events. All experiments were repeated at least three times

### mRNA microinjection

The human full-length NRP-1 and a construct with the last three amino acids deleted (S-E-A), the human full-length GIPC and its PDZ domain deletion mutant were each cloned into the pCS2 vector respectively. The transcribed mRNAs were injected together with the NRP-1 morpholino or alone into 1-4 cell staged embryos. By using a splicing donor morpholino, it is possible to reduce the endogenous expression of the gene, without disturbing the expression of ectopic mRNAs. All experiments were repeated at least three times.

### Antibody staining

4% paraformaldehyde-fixed zebrafish embryos were permeabilized with Proteinase K, then incubated with anti-GFP antibody at 4°C overnight. After washing with PBST, the zebrafish embryos were used for TUNEL staining.

### Detection of apoptotic EC in zebrafish

To examine the functions of NRP-1 in zebrafish EC apoptosis, morpholinos were injected into a Fli-GFP transgenic fish line, where all of the EC are labeled with GFP. Then, zebrafish embryos were fixed at different time points after injection (24, 30, 36, 42 and 48 hpf) with a freshly prepared fixation solution (4% PFA) overnight at 4°C. Antibody-stain with anti-GFP antibody was performed to increase the fluorescence green signal in vessel that has been reduced due to fluorescence diffusion by fixation. Apoptosis was characterized with similar steps as described above using the In Situ Cell Death Detection Kit, TMR red (Roche). Antibody-stained embryos were incubated with the reaction mixture directly. Imaging of blood vessels in TG(fli1:egfp)y1 embryos was performed using a Zeiss confocal laser microscope. Transmitted light images were obtained with a Leica MZ FLIII microscope equipped with a Nikon COOLPIX8700 digital camera. The identity of the apoptotic cells was confirmed by using the red channel where EC would be labeled by Fli-GFP signal.

### Quantitative RT-PCR

Total RNA was extracted using Trizol reagent (Invitrogen, Carlsbad, CA) Quantitative RT-PCR was carried out on 200ng RNA using the lightCycler RNA amplification Kit SYBR Green (Roche) in a LightCycler 2.0 Instrument, following the manufacturer's protocols.

## Supporting Information

Figure S1 NRP-1 mediates VPF/VEGF-induced EC survival. NRP-1 mediates VPF/VEGFinduced PAEC survival. TUNEL assay was performed in PAEC or PAEC/NRP-1 stimulated with or withour 10 ng/ml VPF/VEGF for 48 hours. (i) PAEC/5%FBS. (ii) PAEC/0.1% FBS. (iii) PAEC/0.1% FBS/VEGF; (iv) PAEC/NRP-1/5%FBS; (v) PAEC/NRP-1/0.1% FBS; (vi)PAEC/NRP-1/0.1%FBS/VEGF. b. EGNP-1 mediates HUVEC survival; the C-terminal three amino acids of NRP-1 are essential for this function. TUNEL assay was performed in apoptosis-induced HUVEC transfected with EGNP-1 or EGNP-1ΔSEA and stimulated with or without 10 ng/ml EGF for 24 hours. (i) HUVEC/5% FBS. (ii) HUVEC/0.1%FBS. (iii) HUVEC/EGNP-1/0.1%FBS. (iv) HUVEC/LacZ/0.1%FBS/EGF. (v) HUVEC/EGNP-1/0.1% FBS/EGF. (vi) HUVEC/EGNP-1 ΔSEA/0.1%FBS/EGF. c. Determination of the quantity of apoptotic EC nuclei in zebrafish (n>30). Fluorescent image of embryos injected with indicated morpholinos and mRNA, then subjected to the antibody stain with anti-GFP antibody and TUNEL assay to detect apoptosis. i. Control; ii. Mismatch MO (4.5 ng); iii. zNRP-1a/1b MOs (1.5 ng+1.5 ng); iv. zNRP-1a/1b MOs (4.5 ng+4.5 ng); v. zNRP-1a/1b MOs (4.5 ng+4.5 ng)+hNRP-1 mRNA (0.1 ng); vi. zNRP-1a/1b MOs (4.5 ng+4.5 ng)+hNRP-1 mRNA (0.3 ng); vii. zNRP-1a/1b MOs (4.5 ng+4.5 ng)+hNRP-1ΔSEA mRNA (0.1 ng); vii. zNRP-1a/1b MOs (4.5 ng+4.5 ng)+hNRP-1ΔSEA mRNA (0.3 ng). d. Determination of the quantity of vessel defects (n>30) in embryos as in c. e. Determination of the quantity of apoptotic EC nuclei in different stage zebrafish embryos (n>20). Fluorescent image of different stage embryos injected with indicated morpholinos and subjected to the antibody stain and TUNEL assay to detect apoptosis. *p<0.001, **p<0.005, ***p<0.01, ****p<0.05 in a Student's t test.
(4.90 MB TIF)

Figure S2 GIPC is involved in NRP-1-mediated EC survival. a. GIPC is involved in NRP-1-mediated HUVEC survival. TUNEL assay was performed in HUVEC transfected with EGNP-1 and then transfected with siRNA, and stimulated with or without 10 ng/ml GEF for 48 hours. (i) HUVEC/5%FBS. (ii) HUVEC/0.1% FBS. (iii) HUVEC/EGNP-1/0.1% FBS/EGF. (iv) HUVEC/EGNP-1/0.03pM GIPCsiRNA/0.1% FBS/EGF. (v) HUVEC/EGNP-1/0.3pM GIPCsiRNA/0.1% FBS/EGF. (vi) HUVEC/EGNP-1/3pM GIPCsiRNA/0.1% FBS/EGF. (vii) HUVEC/EGNP-1/3pM control siRNA/0.1% FBS/EGF. b. Determination of the quantity of apoptotic EC nuclei in zebrafish (n>30). Fluorescent image of embryos injected with indicated morpholinos and mRNA, then subjected to the antibody stain with anti-GFP antibody and TUNEL assay to detect apoptosis. Insets show a magnification of apoptotic foci in the vessel. The arrows indicate apoptotic EC. i. Control; ii. zGIPC MOs (1.5 ng); iii. zGIPC MO (4.5 ng); iv. zGIPC MO (4.5 ng)+hGIPC mRNA (0.1 ng); v. zGIPC (4.5 ng)+hGIPC mRNA (0.3 ng); vi. zGIPC MO (4.5 ng)+hGIPCΔPDZ mRNA (0.1 ng); vii. zGIPC MO (4.5 ng)+hGIPCΔPDZ mRNA (0.3 ng). c. Determination of the quantity of vessel defects (n>30) in embryos as in b. *p<0.001, **p<0.005, ***p<0.01, ****p<0.05 in a Student's t test.
(1.82 MB TIF)

Figure S3 PI-3K/Akt is Involved in NRP-1-mediated HUVEC survival. a. PI3K is involved in NRP-1-mediated EC survival signaling. Apoptosis assay was performed in HUVEC transfected with EGNP-1 or co-transfected with EGNP-1 with p85(DN) and stimulated with or without 10 ng/ml EGF for 48 hours, and then treated with LY294002 for 39 minutes, accordingly. (i) HUVEC/5%FBS. (ii) HUVEC/0.1%FBS. (iii) HUVEC/EGNP-1/EGF. (iv) HUVEC/LacZ/EGF. (v) HUVEC/EGNP-1+ 25μMLy294002/EGF. (vi) HUVEC/EGNP-1, p85(DN)/EGF. (vii) HUVEC/EGNP-1, p110CAAX/EGF. b. Akt is involved in PI3K-mediated NRP-1 survival signaling in EC. Apoptosis assays were performed in HUVEC transfected with EGNP-1 or cotransfected EGNP-1 with Akt(DN) or Akt(Active) and stimulated with or without 10 ng/ng EGF for 48 hours . (i) HUVEC/5% FBS. (ii) HUVEC/0.1%FBS. (iii) HUVEC/EGNP-1/0.1%FBS/EGF. (iv) HUVEC/EGNP-1, LacZ/0.1%FBS/EGF. (v) HUVEC/EGNP-1, Akt(DN)/0.1%FBS/EGF. (vi) HUVEC/EGNP-1, Akt(Active)/0.1% FBS/EGF.
(4.46 MB TIF)

Figure S4 p53 inactivation in NRP-1-mediated EC survival. a. Determination of the quantity of apoptotic EC nuclei in zebrafish embryos (n>30). Fluorescent image of embryos injected with indicated morpholinos and subjected to the antibody stain with anti-GFP antibody and TUNEL assay to detect apoptosis. i. Control; ii. zNRP-1a/1b MOs (4.5 ng+4.5 ng); iii. zNRP- 1a/1b MOs (4.5 ng+4.5 ng)+zp53 MO (4.5 ng); vi. zNRP-1a/1b MOs (4.5 ng+4.5 ng)+zp53 MO (9.0 ng); v. zMdm2 MO (4.5 ng); vi. zMdm2 MO (4.5 ng)+zp53 MO (4.5 ng); vii. zMdm2 MO (4.5 ng)+zp53 MO (9.0 ng). b. Determination of the quantity of vessel defects (n>30) in embryos as in b. *p<0.001, **p<0.005, ***p<0.01, ****p<0.05 in a Student's t test.
(0.17 MB TIF)
