# Supplementary figures and images for: Neuropilin-1 Modulates p53/Caspases Axis to Promote Endothelial Cell Survival
Source: PLoS One. 2007 Nov 14;2(11):e1161. doi: 10.1371/journal.pone.0001161 (PMC2048754; doi:10.1371/journal.pone.0001161)

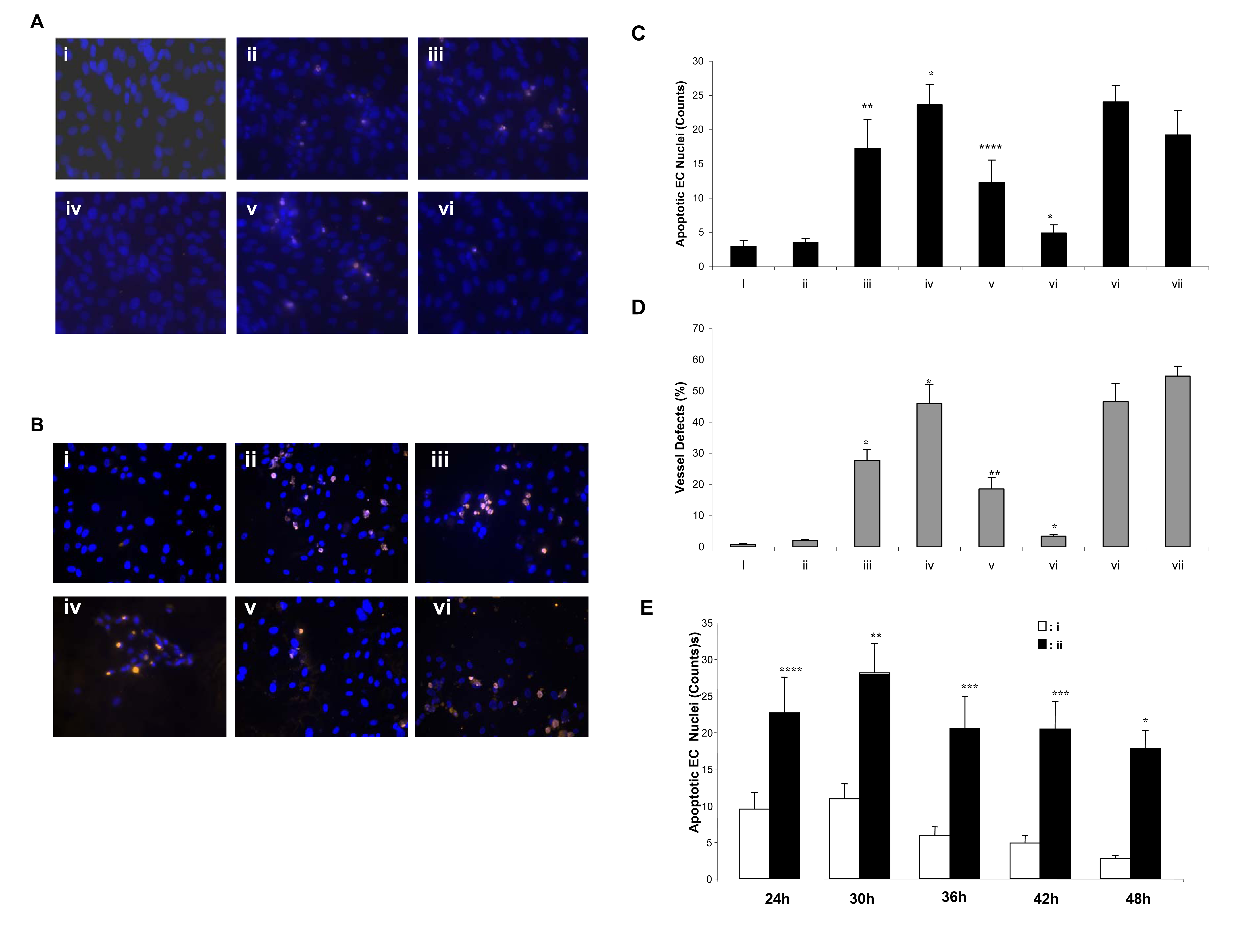

Supplement: Figure S1 — NRP-1 mediates VPF/VEGF-induced EC survival. NRP-1 mediates VPF/VEGFinduced PAEC survival. TUNEL assay was performed in PAEC or PAEC/NRP-1 stimulated with or withour 10 ng/ml VPF/VEGF for 48 hours. (i) PAEC/5%FBS. (ii) PAEC/0.1% FBS. (iii) PAEC/0.1% FBS/VEGF; (iv) PAEC/NRP-1/5%FBS; (v) PAEC/NRP-1/0.1% FBS; (vi)PAEC/NRP-1/0.1%FBS/VEGF. b. EGNP-1 mediates HUVEC survival; the C-terminal three amino acids of NRP-1 are essential for this function. TUNEL assay was performed in apoptosis-induced HUVEC transfected with EGNP-1 or EGNP-1ΔSEA and stimulated with or without 10 ng/ml EGF for 24 hours. (i) HUVEC/5% FBS. (ii) HUVEC/0.1%FBS. (iii) HUVEC/EGNP-1/0.1%FBS. (iv) HUVEC/LacZ/0.1%FBS/EGF. (v) HUVEC/EGNP-1/0.1% FBS/EGF. (vi) HUVEC/EGNP-1 ΔSEA/0.1%FBS/EGF. c. Determination of the quantity of apoptotic EC nuclei in zebrafish (n>30). Fluorescent image of embryos injected with indicated morpholinos and mRNA, then subjected to the antibody stain with anti-GFP antibody and TUNEL assay to detect apoptosis. i. Control; ii. Mismatch MO (4.5 ng); iii. zNRP-1a/1b MOs (1.5 ng+1.5 ng); iv. zNRP-1a/1b MOs (4.5 ng+4.5 ng); v. zNRP-1a/1b MOs (4.5 ng+4.5 ng)+hNRP-1 mRNA (0.1 ng); vi. zNRP-1a/1b MOs (4.5 ng+4.5 ng)+hNRP-1 mRNA (0.3 ng); vii. zNRP-1a/1b MOs (4.5 ng+4.5 ng)+hNRP-1ΔSEA mRNA (0.1 ng); vii. zNRP-1a/1b MOs (4.5 ng+4.5 ng)+hNRP-1ΔSEA mRNA (0.3 ng). d. Determination of the quantity of vessel defects (n>30) in embryos as in c. e. Determination of the quantity of apoptotic EC nuclei in different stage zebrafish embryos (n>20). Fluorescent image of different stage embryos injected with indicated morpholinos and subjected to the antibody stain and TUNEL assay to detect apoptosis. *p<0.001, **p<0.005, ***p<0.01, ****p<0.05 in a Student's t test. (4.90 MB TIF) [file pone.0001161.s001.tif]

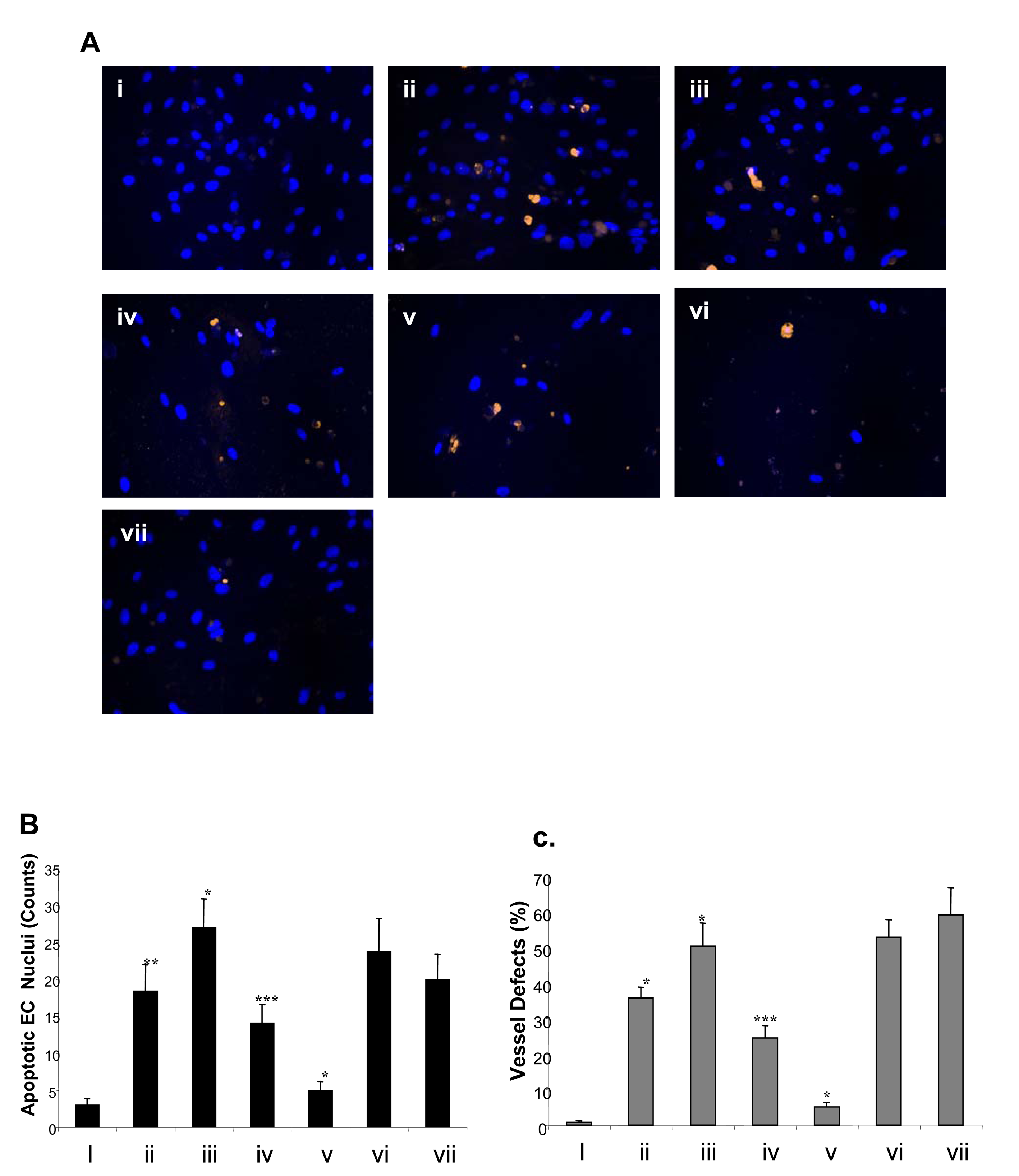

Supplement: Figure S2 — GIPC is involved in NRP-1-mediated EC survival. a. GIPC is involved in NRP-1-mediated HUVEC survival. TUNEL assay was performed in HUVEC transfected with EGNP-1 and then transfected with siRNA, and stimulated with or without 10 ng/ml GEF for 48 hours. (i) HUVEC/5%FBS. (ii) HUVEC/0.1% FBS. (iii) HUVEC/EGNP-1/0.1% FBS/EGF. (iv) HUVEC/EGNP-1/0.03pM GIPCsiRNA/0.1% FBS/EGF. (v) HUVEC/EGNP-1/0.3pM GIPCsiRNA/0.1% FBS/EGF. (vi) HUVEC/EGNP-1/3pM GIPCsiRNA/0.1% FBS/EGF. (vii) HUVEC/EGNP-1/3pM control siRNA/0.1% FBS/EGF. b. Determination of the quantity of apoptotic EC nuclei in zebrafish (n>30). Fluorescent image of embryos injected with indicated morpholinos and mRNA, then subjected to the antibody stain with anti-GFP antibody and TUNEL assay to detect apoptosis. Insets show a magnification of apoptotic foci in the vessel. The arrows indicate apoptotic EC. i. Control; ii. zGIPC MOs (1.5 ng); iii. zGIPC MO (4.5 ng); iv. zGIPC MO (4.5 ng)+hGIPC mRNA (0.1 ng); v. zGIPC (4.5 ng)+hGIPC mRNA (0.3 ng); vi. zGIPC MO (4.5 ng)+hGIPCΔPDZ mRNA (0.1 ng); vii. zGIPC MO (4.5 ng)+hGIPCΔPDZ mRNA (0.3 ng). c. Determination of the quantity of vessel defects (n>30) in embryos as in b. *p<0.001, **p<0.005, ***p<0.01, ****p<0.05 in a Student's t test. (1.82 MB TIF) [file pone.0001161.s002.tif]

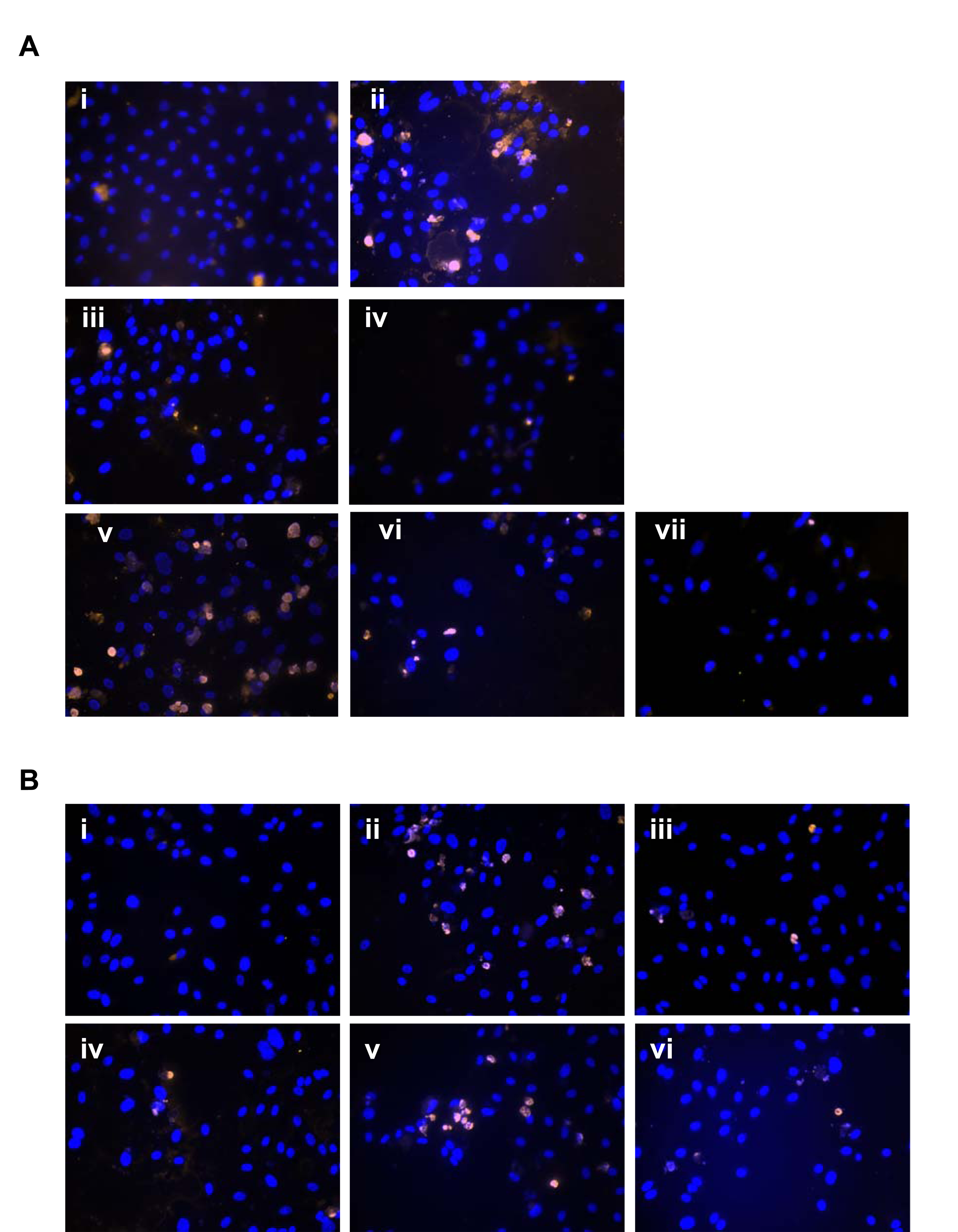

Supplement: Figure S3 — PI-3K/Akt is Involved in NRP-1-mediated HUVEC survival. a. PI3K is involved in NRP-1-mediated EC survival signaling. Apoptosis assay was performed in HUVEC transfected with EGNP-1 or co-transfected with EGNP-1 with p85(DN) and stimulated with or without 10 ng/ml EGF for 48 hours, and then treated with LY294002 for 39 minutes, accordingly. (i) HUVEC/5%FBS. (ii) HUVEC/0.1%FBS. (iii) HUVEC/EGNP-1/EGF. (iv) HUVEC/LacZ/EGF. (v) HUVEC/EGNP-1+ 25μMLy294002/EGF. (vi) HUVEC/EGNP-1, p85(DN)/EGF. (vii) HUVEC/EGNP-1, p110CAAX/EGF. b. Akt is involved in PI3K-mediated NRP-1 survival signaling in EC. Apoptosis assays were performed in HUVEC transfected with EGNP-1 or cotransfected EGNP-1 with Akt(DN) or Akt(Active) and stimulated with or without 10 ng/ng EGF for 48 hours . (i) HUVEC/5% FBS. (ii) HUVEC/0.1%FBS. (iii) HUVEC/EGNP-1/0.1%FBS/EGF. (iv) HUVEC/EGNP-1, LacZ/0.1%FBS/EGF. (v) HUVEC/EGNP-1, Akt(DN)/0.1%FBS/EGF. (vi) HUVEC/EGNP-1, Akt(Active)/0.1% FBS/EGF. (4.46 MB TIF) [file pone.0001161.s003.tif]

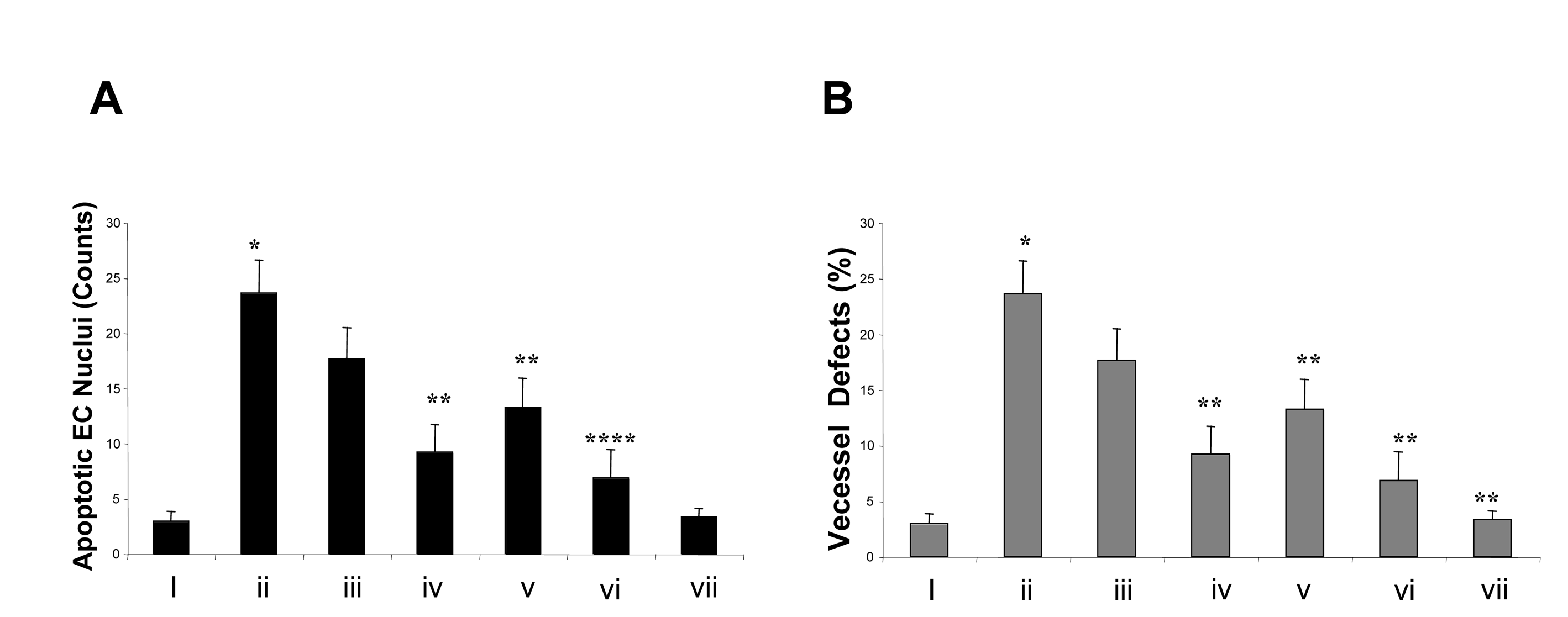

Supplement: Figure S4 — p53 inactivation in NRP-1-mediated EC survival. a. Determination of the quantity of apoptotic EC nuclei in zebrafish embryos (n>30). Fluorescent image of embryos injected with indicated morpholinos and subjected to the antibody stain with anti-GFP antibody and TUNEL assay to detect apoptosis. i. Control; ii. zNRP-1a/1b MOs (4.5 ng+4.5 ng); iii. zNRP- 1a/1b MOs (4.5 ng+4.5 ng)+zp53 MO (4.5 ng); vi. zNRP-1a/1b MOs (4.5 ng+4.5 ng)+zp53 MO (9.0 ng); v. zMdm2 MO (4.5 ng); vi. zMdm2 MO (4.5 ng)+zp53 MO (4.5 ng); vii. zMdm2 MO (4.5 ng)+zp53 MO (9.0 ng). b. Determination of the quantity of vessel defects (n>30) in embryos as in b. *p<0.001, **p<0.005, ***p<0.01, ****p<0.05 in a Student's t test. (0.17 MB TIF) [file pone.0001161.s004.tif]
